# Targeting lipid reprogramming in the tumor microenvironment by traditional Chinese medicines as a potential cancer treatment

**DOI:** 10.1016/j.heliyon.2024.e30807

**Published:** 2024-05-07

**Authors:** Qian Zuo, Yingchao Wu, Yuyu Hu, Cui Shao, Yuqi Liang, Liushan Chen, Qianqian Guo, Ping Huang, Qianjun Chen

**Affiliations:** a State Key Laboratory of Traditional Chinese Medicine Syndrome, The Second Affiliated Hospital of Guangzhou University of Chinese Medicine, Guangzhou, China; b Department of Breast, Guangdong Provincial Hospital of Chinese Medicine, Guangzhou, China; c Guangdong Academy of Traditional Chinese Medicine, Guangzhou, China; d The First Affiliated Traditional Chinese Medicine Hospital of Guangzhou Medical University, Guangzhou, China

**Keywords:** Tumor microenvironment, Lipid reprogramming, Traditional Chinese medicines, Fatty acids, de novo lipogenesis, Lipid droplet

## Abstract

In the last ten years, there has been a notable rise in the study of metabolic abnormalities in cancer cells. However, compared to glucose or glutamine metabolism, less attention has been paid to the importance of lipid metabolism in tumorigenesis. Recent developments in lipidomics technologies have allowed for detailed analysis of lipid profiles within cancer cells and other cellular players present within the tumor microenvironment (TME). Traditional Chinese medicine (TCM) and its bioactive components have a long history of use in cancer treatments and are also being studied for their potential role in regulating metabolic reprogramming within TME. This review focuses on four core abnormalities altered by lipid reprogramming in cancer cells: de novo synthesis and exogenous uptake of fatty acids (FAs), upregulated fatty acid oxidation (FAO), cholesterol accumulation, which offer benefits for tumor growth and metastasis. The review also discusses how altered lipid metabolism impacts infiltrating immune cell function and phenotype as these interactions between cancer-stromal become more pronounced during tumor progression. Finally, recent literature is highlighted regarding how cancer cells can be metabolically reprogrammed by specific Chinese herbal components with potential therapeutic benefits related to lipid metabolic and signaling pathways.

## 1 Introduction

Since the first serious discussions and analyses of metabolic alterations in tumors emerged nearly a century ago [1], more recent attention has focused on the field of cancer metabolism in the past decades. Nowadays, several lines of evidence suggest that tumor cells possess a unique metabolic profile and undergo tumor-related changes in metabolism at different stages of tumorigenesis [2,3]. To support uncontrolled cell proliferation, excessive intake of nutrients is required from the environment to meet its biosynthetic needs [4]. Besides the Warburg effect and heightened glutaminolysis, cancer metabolic reprogramming relies on lipid metabolism [5]. Lipids play a crucial role in the development and progression of malignant cells. Specifically, fatty acids, phospholipids, cholesterol, and triglycerides are involved in various biological processes such as energy storage, membrane biosynthesis, and cellular signaling transduction [6]. Altered levels of lipids and saturated/unsaturated fatty acid can lead to uncontrolled homeostasis and enhanced cellular stress [7,8]. In comparison to glucose and amino acids metabolism, however, there is a relatively small body of literature highlighting the alteration in lipid metabolism and signaling as the hallmarks of aberrant cell growth and cancer progression.

Lipid metabolism disorders in cancer including multiple patterns, such as FAO, cholesterol accumulation, out-of-controlled lipid uptake, and endogenous de novo FAs synthesis, thereby facilitating tumor growth and progression. Moreover, tumor cells with high proliferation demonstrate a significant affinity for lipids and cholesterol, satisfied by either facilitating synthesis of cholesterol and lipogenesis, or accelerating uptake of exogenous (or dietary) lipids and lipoproteins. Lipid droplets (LDs) are consist of accumulated lipids and cholesterol. Meanwhile, elevated levels of LDs and cholesteryl ester accumulation within tumors are currently recognized as indicators of cancer aggressiveness [[9], [10], [11]]. Similarly, LD-rich cancer cells can regulate cell proliferation via offering extra substrates for cell growth or altering cell signaling pathway. Recent data suggests that lipid metabolism is crucial in creating a pro-tumor microenvironment. This indicates the need to investigate not only cancer cells but also immune and stromal cells in relation to obesity [[12], [13], [14]]. The literature on lipid metabolism in pan-cancer shows that metabolic changes affect all cell types and their interactions within the TME. The acquirements of how different types of cells affect the lipid metabolism of cancer cells or react to changes in lipid levels is crucial for developing effective therapeutic strategies.

TCM has been extensively applied alone or as an alternative approach for various diseases, including cancer, over thousands of years in China [15]. Growing evidence has emphasized the clear benefits of TCM compared to traditional therapies in regards to reduced side effects, lower drug toxicity, and decreased economic costs [16,17]. Relying on the significance of lipid metabolic reprogramming at different stages of tumorigenesis, lipid metabolism would developed as an attractive direction for cancer treatments. Nevertheless, the intricacies of the possible mechanisms underlying metabolic pathways hinder current methods for drug discovery that focus on finding individual drugs that interact with clearly defined molecular targets (such as a single receptor or enzyme). Instead, there is growing interest in the “multi-component and multi-target” effects of traditional Chinese medicines. It has been discovered that multiple disorders respond better to therapies involving multiple drugs or targets, which could lead to new approaches for cancer treatment from a metabolic standpoint [18,19]. In this review, we analyze the specific characteristics of lipid metabolic reprogramming associated with tumorigenesis and investigate how these characteristics contribute to the development and establishment of a tumorigenic state. With this focus, we summarized recent advances of both regulatory effects and molecular mechanisms of TCM in rewiring lipid metabolism. We propose that lipid metabolism is emerging as a promising anticancer therapy as cancer cells heavily rely on it to acquire energy to maintain cellular homeostasis [20]. Given the significance of lipid metabolism to cancers, investigating Chinese herbal interference in cancer lipid reprogramming might identify new therapeutic targets for cancer and broaden the strategies for managing metabolism-associated disorders.

## 2 Regulatory effects of lipid reprogramming within the tumor cells facilitate cancer progression

**Fig. 1:**
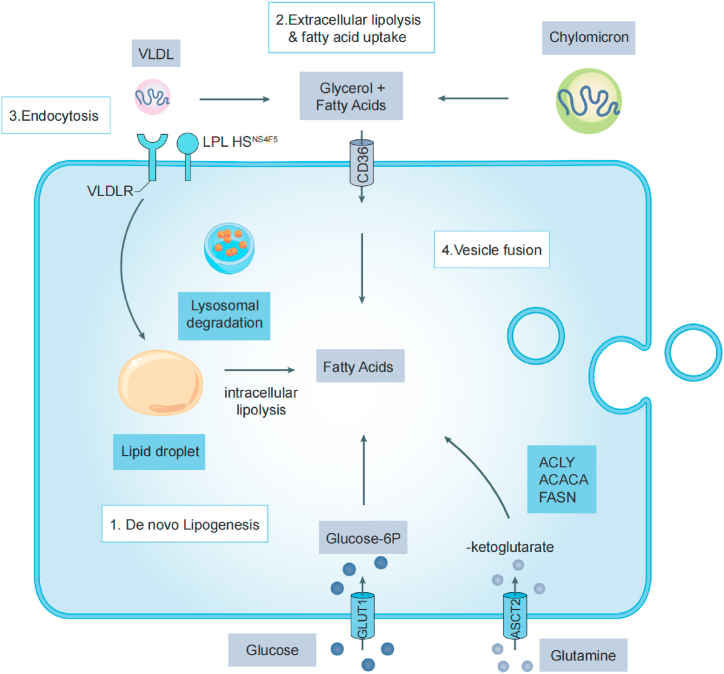
Currently recognized mechanisms of lipid acquisition by cancer cells.

Lipid metabolism deregulation in cancer involves various factors, such as the production of new fatty acids and cholesterol, increased absorption of lipids, heightened FAO activity, and the development of cancer-associated adipose tissue. Furthermore, alterations in lipid metabolism can also impact immune cells through metabolic competition, onco-metabolites, and exosomes [21,22]. This section primarily focuses on recent advancements in FA, cholesterol and lipid metabolism within tumor cells and their contribution to rapid proliferation and invasiveness (Fig. 1). We also emphasize the distinct function of lipids in the microenvironment that goes beyond their metabolic necessities (Table 1).

**Table 1:** Lipids and metabolic pathways influencing tumor progression.

metabolic pathway implicated	Lipid protein	Impact on cancer	Reference
lipid uptake	LPL	invasiveness capability	[[23], [24], [25]]
	CD36	tumor formation, growth, metastasis, enhanced chemoresistance and radio-resistance, disrupted anti-tumor Immune responses	[[26], [27], [28], [29]]
de novo FAs synthesis	FASN	cancer progression	[[30], [31], [32], [33]]
fatty acid oxidation	CPT	cancer proliferation, survival, drug resistance, and stemness	[[34], [35], [36], [37], [38]]
cholesterol biosynthesis	SREBP	tumorigenesis	[39,40]
abnormal distribution of cholesterol	ABCs, ACATs	reduces apoptosis and promotes cell cycle progression	[[41], [42], [43]]
lipid droplet metabolism	Lipin 1	foster tumor growth and aggressiveness	[44]

### 2.1 Reprogrammed fatty acid metabolism in cancer cells

The metabolism of fatty acids (FA) involves several steps, including lipid uptake, de novo FA synthesis, and FAO. Numerous studies have demonstrated the influence of exogenous free fatty acid (FFA) uptake on tumor progression across various types of cancer, including melanoma [45], gastric [46], ovarian [47], prostate [48], and colon cancers [49]. Literature has addressed the role of lipoprotein lipase (LPL), CD36, fatty acid transport proteins (FATPs), and fatty acid-binding proteins (FABPs) in lipolysis [23,24]. The enzyme LPL, responsible for breaking down triacylglycerols (TGs) and very-low-density lipoprotein (VLDL), is overexpressed in invasive cervical squamous cell carcinomas [25]. Recently, CD36 has been concluded by increasing studies that it contributes to promoted tumor formation, growth, metastasis, enhanced chemoresistance and radio-resistance, disrupted anti-tumor Immune responses, etc. [[26], [27], [28]]. Besides, accumulated studies reported the aberrant expression of CD36 and LPL in cancer cell line [29]. Collectively, these studies indicate that cancer cells use fatty acids obtained from the diet through lipolysis in the bloodstream to support their growth.

In addition to increased lipid uptake, enhanced de novo FAs synthesis has been extensively regarded as a hallmark of several cancer cells [6,30,50]. Cancer cells activate lipogenic enzymes, including ATP-citrate lyase (ACLY), acetyl-CoA carboxylase (ACC), CoA carboxylase (ACACA), fatty acid synthase (FASN), and SCD, resulting in increased production of fatty acids [30]. Although FASN expression remains low in most normal human tissues, it has been observed to be significantly elevated in several cancer types such as breast, prostate, colon and lung cancers [[30], [31], [32]]. Recent studies have shown that FASN is a metabolic feature of prostate cancer progression, particularly in metastatic castration-resistant prostate cancer (mCRPC) [33]. It is worth mentioning that cancer cells exhibit increased FAO level and educate a pro-tumor microenvironment [34]. Similarly, cancer cells exposed to acidosis for a prolonged period of time manifest induced FAO activities [51]. And the rate-limiting enzyme for FAO, carnitine palmitoyltransferase I (CPT1), has been found to promote cancer proliferation, survival, drug resistance, and stemness [[34], [35], [36]]. In colon cancer, overexpression of CPT2 was significantly correlated with primary tumor stage and better prognosis [37]. More recently, it has been demonstrated that the downregulation of CPT2 is highly significant in predicting hepatocellular carcinoma (HCC) [38]. Above all, dysregulated lipid metabolism is a significant metabolic alteration in cancer, highlighting the potential of specific metabolites as prognostic/diagnostic indicators for cancers.

### 2.2 Deregulation of cholesterol metabolism in cancer cells

Cholesterol plays regulative roles in both fluidity and flexibility of the membrane. Maintaining cholesterol homeostasis involves not only synthesizing new cholesterol and absorbing it from the diet but also processes such as reverse cholesterol transport (RCT), bile acid synthesis, and biliary excretion of cholesterol [52]. Since some reports have demonstrated that certain type of cancer are highlighted with increased cholesterol biosynthesis, such as prostate cancer, breast cancer, and HCC, some proteins involved in cholesterol metabolism have been accordingly characterized as cancer-related factors [53]. The biosynthesis of cholesterol, which is mediated by sterol regulatory element-binding protein (SREBP), plays a vital role in maintaining cholesterol homeostasis via activation of PI3K/AKT and RAS/MAPK signaling pathways. Additionally, SREBP2 acts as the primary transcription factor that triggers the activation of genes linked to mevalonate and cholesterol synthesis [39]. In breast cancer cells, mutated p53 - a frequent target for mutations in tumors - causes an excessive response in the mevalonate pathway by recruiting numerous sterol biosynthesis genes or potentially through one or more of the SREBP proteins [40].

In addition to the enhanced de novo lipogenesis, abnormal distribution of cellular cholesterol is highlighted as a cancer-associated factor. This implies that cancer cells require more cholesterol, which is often accompanied by an increased intake of cholesterol from low-density lipoproteins (LDL) and the overexpression of the LDL receptor in many types of cancer [54]. In addition, cells export excess cholesterol using ATP-binding cassette (ABC) transporters such as ATP Binding Cassette Subfamily A Member 1 (ABCA1) and G subfamily of ABC transporters in mammals (ABCGs). Alternatively, as the excess cholesterol can be converted to less toxic cholesteryl esters (CEs) by acy-lCoA-cholesterol acyltransferases (ACATs), the promoted CE levels were frequently observed in various types of cancer [[41], [42], [43]]. The activation of PKA/SUFU/GLI1 signaling through the smoothened receptor, also known as Hedgehog signaling, induced by cholesterol reduces apoptosis and promotes cell cycle progression [55].

### 2.3 Alterations of lipid droplet metabolism in cancer cells

LDs are formed in the endoplasmic reticulum (ER) and have a neutral lipid core surrounded by a phospholipid monolayer [56]. Cancer cells use lipid droplets to maintain energy production, redox balance, regulate autophagy, facilitate membrane synthesis and control its composition, which helps in reducing stress and promoting tumor progression [57]. In addition to utilization as important metabolic organelles in maintaining the stability of the intracellular environment, lipid droplets can play multiple roles in the cancer cells [58]. Lipid-rich tumors have been linked to a high aggressive potential and poor clinical outcomes [10,59]. Previous studies reported that increased lipid droplets in breast and ovarian cancer cells were attributed to the neighboring adipocytes, implying that adipocytes-derived FAs sustains metabolic reprogramming in cancer cells and foster tumor aggressiveness [60,61]. Additionally, recent studies in kidney cancer have identified the significance of lipid droplets in maintaining membrane saturation and organelle homeostasis during hypoxia [62,63]. Clear-cell renal cell carcinoma (ccRCC) is characterized by its lipid-rich cytoplasmic deposits and consistent hypoxia-inducible factor (HIF) signaling activation. Similarly, HCC and cervical adenocarcinoma cells can accumulate lipid droplets when exposed to hypoxia, which is achieved by stimulating the expression of phosphatidate phosphatase-1 (Lipin 1) in a HIF-1α-dependent manner [44]. Clearly, dysregulated lipid droplet turnover are key players in cancer cells and tissues, which provide clues for drug development in the future.

There is no doubt that lipid metabolism and lipid synthesis are closely related to hormone-related cancers. Studies have shown that melatonin is able to damage the mitochondrial membrane and increase lipid bodies and autophagic vacuole formation, which trigger cell cycle arrest and induce liver cancer cell death by apoptosis [64]. Knockout of androgen receptors in prostate cancer cells significantly affected the transcription of genes involved in fatty acids, cholesterol and sterol biosynthesis, as well as to lipid and fatty acid transportation pathways [65]. Metabolomic analysis of breast cancer patients showed that estrogen receptor positive tumors contain elevated levels of short and medium chain fatty acids, as well as increased carnitine derivative levels, compared to estrogen receptor negative, suggesting an elevated fatty acid transportation rate [66]. These data suggest that hormones may play a role in tumor by regulating lipid metabolism in cancer cell. Therefore, they are promising therapeutic targets.

## 3 Regulatory effects of lipid reprogramming within the immune cells in tumor microenvironment facilitate cancer progression

### 3.1 T cells

**Fig. 2:**
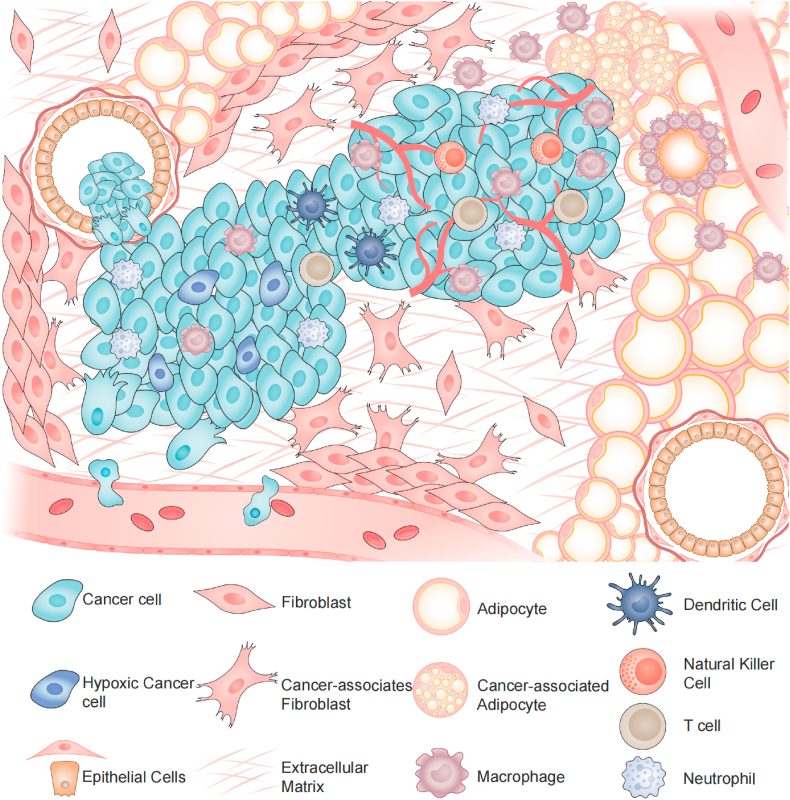
Complex constitution within the tumor microenvironment (TME).

The immune cells within the tumor microenvironment (TME) play important roles in tumorigenesis (Fig. 2). As key players in anti-tumor immunity, T cells can be classified into various subtypes and have the ability to travel through blood vessels to reach tumors. Once they infiltrate the tumor, they are capable of recognizing cancer cells and eliminating them. CD4^+^ T cells can be categorized as regulatory T (Treg) cells or conventional T helper (Th) cells. On the other hand, CD8^+^ effector T cells are also known as cytotoxic T cells (Tc). These mature CD8^+^ T cells play a crucial role in identifying infected, damaged, or cancerous somatic cells and initiating death pathways through cytotoxic proteins. CD8^+^ T cell infiltration within TME shows close correlations with positive patient outcomes. Thus, T-cell infiltration might serve as a tool to monitor the tumor growth and progression, which displays great therapeutic potentials [67].

Emerging evidence has implied that T cells exhibit lipid metabolism flexibility and adapt to their surrounding nutrient environments, driving the antitumor or pro-tumor immunity in specific TME states [68,69]. This is especially important for kidney, stomach, breast, and colon cancers that tend to thrive in environments with high levels of fat [61,69,70]. As two important signaling pathways of lipids homeostasis, liver X receptors (LXR) signaling serves as a suppressive role in T cell activation, whereas SREBP2 signaling facilitates the proliferation and effector function of T cells [71]. By inhibiting Acetyl-CoA Acetyltransferase 1 (ACAT1) in CD8^+^ T cells, the synthesis of cholesterol is altered, resulting in an increase of free cholesterol levels on the plasma membrane. This increase leads to the formation of immunological synapses that effectively target and kill tumor cells [72]. Another study reported that avasimibe, an ACAT1 inhibitor, could strengthen the antitumor effect of chimeric antigen receptor (CAR)-T cells by triggering cytotoxic CD8^+^ T cell infiltration in tumor sites [73]. Recently, cholesterol accumulation has been shown to trigger T cell exhaustion via enhanced endoplasmic reticulum stress (ER stress) [74]. Forkhead Box P3 (FoxP3) is a crucial regulator of immune regulatory T cells (Tregs) gene expression and is responsible for Tregs development and function, involved in FAs uptake, Oxidative phosphorylation (OXPHOS), and FAO. According to a recent study, Tregs-rich TME was not only highly suppressive but also displayed accelerated lipid biosynthesis via FAO [75]. Despite the debate surrounding the significance of fatty acids and their breakdown, their impact on enhancing or inhibiting CD8^+^ T cell effector functions is context dependent. Given their effects on T cells, it is still necessary to conduct further studies to determine whether fatty acids can be used as metabolic-based therapies for eradicating tumors.

### 3.2 Tumor-associated macrophages and dendritic cells

As the major cell population recruited to the tumor site, macrophages have been involved at all stages of tumor progression [76]. Infiltrating macrophages can be classified into two phenotypes - M1 macrophages, which have a pro-inflammatory phenotype and are capable of killing pathogens, and M2 macrophages, which promote cancer cell proliferation and tissue repair. Previous literature has shown the complexities in the properties and function of macrophages subtypes [77]. Tumor-associated macrophages (TAMs) exhibiting M2-like characteristics are known to have a significant impact on tumor growth and metastasis [15,78], angiogenesis [79,80], extracellular matrix modification [81], as well as inhibition of T cell activation and effector functions against cancer cells [82]. To date, multiple studies have identified the positive correlations between the presence of M2-like TAMs and elevated tumor growth and relapse, low tumor response, and poor overall survival [[83], [84], [85]]. Recent investigations have identified cholesterol metabolism as a crucial regulator of macrophage function [86]. It has been observed that in response to hyaluronan oligomers secreted by cancer cells, TAMs enhance the import of cholesterol while decreasing its biosynthesis, leading to the transformation of recruited macrophages into M2-like phenotype. Additionally, TAMs are activated by FASN and peroxisome proliferator-activated receptor β/δ (PPARβ/δ), induced by macrophage colony-stimulating factor (M-CSF) secreted from cancer cells, which further promotes tumor progression [87].

Dendritic cells (DCs) are a type of antigen-presenting cell (APC) that play a crucial role in initiating adaptive immune responses. They act as the “sentinels” of the immune system, responsible for detecting and responding to foreign invaders. By exploring dysfunction within DCs, we may gain insight into why immune cell responses are often ineffective in the tumor microenvironment (TME). Recently, activation of liver X receptor (LXR)α has been shown to interfere with CCR7 expression in DCs, and reduced CCR7-bearing dendritic cells results in restrained migration of dendritic cells to regional lymph nodes, thereby limiting the presentation of tumor antigens to T cells. In tumor-bearing mice, inhibiting LXR-α signaling restores dendritic cell function and antitumor response by reducing cholesterol synthesis [88]. Similarly, lipid accumulation causes DC dysfunction in terms of reduced antigen presentation and poor stimulation of T cell responses [[89], [90], [91]]. A recent study on colorectal cancer found that the accumulation of lipid droplets, which is dependent on lysophosphatidylcholine acyltransferase 2 (LPCAT-2), prevented calreticulin from being exposed to the plasma membrane. This resulted in the inhibition of antigen presentation by dendritic cells (DCs) through MHCII and activation of CD8^+^ T cells [92].

### 3.3 Other immunosuppressive cells

Neutrophils, the most prevalent type of white blood cell in the body, play a vital role in combating inflammation and infections. They are also associated with poor prognosis in cancer patients, as tumor-associated neutrophils (TANs) can promote tumor growth through their metabolic activity [93]. The recruitment of TANs is regulated by the tumor microenvironment (TME) [94,95]and their activation has been linked to oxidized lipid accumulation in ovarian and lung cancer cells, which triggers the fibroblast growth factor-related signaling pathway and leads to early recurrence [96]. In glucose-deficient TME, neutrophils switch from the routine glycolysis to FAO, activating reactive oxygen species (ROS) production and reducing T cell immune responses, and thus contributing to tumorigenesis through immune suppression [97,98]. Furthermore, this has been observed in myeloid-derived suppressor cells that share similar morphology (MDSCs).

**Fig. 3:**
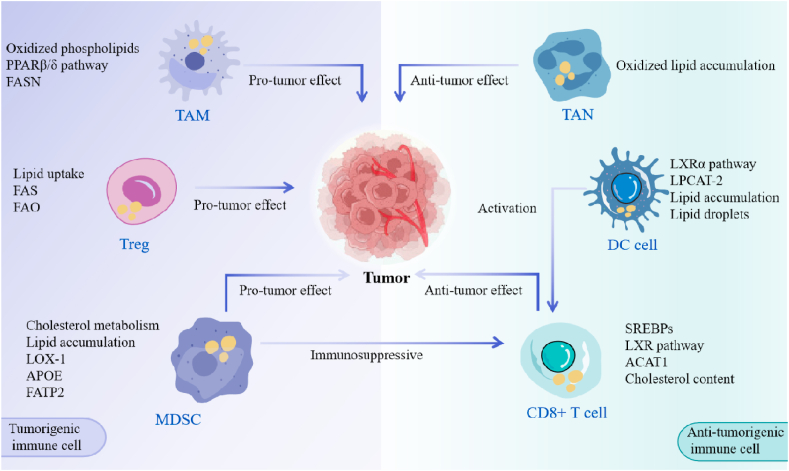
Reprogrammed lipid metabolism interferes with immune cells.

MDSCs expand during pathological conditions in both humans and mice, and they perform similar functions in terms of immune suppression [99]. LOX-1, a lectin-like receptor for oxidized low-density lipoprotein and also an LDL receptor, serves as a distinctive marker to differentiate MDSCs from neutrophils. Additionally, overexpression of LOX-1 indicates altered cholesterol metabolism in MDSCs within TME [100]. Recent studies reported that LXR agonist RGX-104 was able to strongly reduce MDSC numbers and enhance T cell activity via upregulation of Apolipoprotein E (APOE) [101,102]. Moreover, MDSCs in mice with tumors have higher levels of lipid accumulation due to the overexpression of fatty acid transporter 2 (FATP2). The removal of FATP2 eliminates the suppressive function of MDSCs by preventing the absorption of arachidonic acid and inhibiting prostaglandin E2 synthesis [103]. Collectively, understanding the importance of lipid metabolites in shape the immune response within the TME and how these metabolites can be targeted to be a novel avenue for therapeutic strategy (Table 2 and Fig. 3).

**Table 2:** Reprogrammed lipid metabolism interferes with immune cells.

Immune cell type	Lipid species, protein, or metabolic pathway involved	Main observations	Reference
CD4^+^ T cells	Lipid biosynthesis	Tregs-rich TME displayed accelerated lipid biosynthesis via FAO	[75]
CD8^+^ T cells	Cholesterol metabolism, CAT1,	The synthesis of cholesterol in CD8^+^ T cells leads to the formation of immunological synapses, the cytotoxic T cell infiltration in tumor sites, and T cell exhaustion	[[71], [72], [73], [74]]
TAMs	Cholesterol metabolism, FASN and PPARβ/δ,	The import of cholesterol leads to the transformation of recruited macrophages into M2-like phenotype	[[76], [77], [78],^86^,^87^]
Dendritic Cells	LXRα, LPCAT-2, lipid accumulation, lipid droplets	The activation of liver X receptor (LXR)α, LPCAT-2, and lipid accumulation in dendritic cells limit the presentation of tumor antigens to T cells	[[88], [89], [90], [91], [92]]
TANs	Oxidized lipid accumulation	The recruitment of TANs triggers the fibroblast growth factor-related signaling pathway, leads to early recurrence and contributes to tumorigenesis through immune suppression	[[93], [94], [95], [96], [97], [98]]
MDSCs	Cholesterol metabolism, lipid accumulation, LOX-1, APOE, FATP2	The activation of LOX-1, APOE, or FATP2 leads to a increasing number of MDSCs differentiated from neutrophils and immune suppression.	[[100], [101], [102], [103]]

## 4 Regulation of traditional Chinese medicine on lipids metabolism

Based on the comprehensive understanding of lipid metabolism within TME established in the previous text, targeting lipid metabolism holds promise as a multi-dimensional approach that can act on both tumor cells and innate immune cells, resulting in improved therapeutic outcomes. Currently, extensive interests in TCM have been reawakened in light of the awareness that metabolic reprogramming is playing an indispensable role in the biology and proliferation of cancer cells. Beyond the significance of glucose and energy metabolisms in tumorigenesis, it has been also observed that lipid metabolism could regulate the synthesis of cellular membranes and the key proliferation signals of cancer cells. Chinese herbal components have been extensively documented for their effects in lipid metabolism disorders. NAFLD, a well-known lipid metabolism disorder, has shown a significant reduction of lipid accumulation in liver under Dioscin treatment [104]. Likewise, berberine has been demonstrated to have anti-hyperlipidemia effect and applied to the therapy for cardiovascular diseases [105]. Curcumin, a natural compound from ginger, has been revealed by amounts of researchers for its potential to regulate lipid and cholesterol metabolism [106]. Given the significant role of lipid reprogramming in tumor onset and progression, interference with lipid metabolism by TCM provide a reference for drug development in tumors.

### 4.1 TCMs decoctions modulate lipid metabolism of cells within tumor microenvironment

Accumulating studies have reported the involvements of lipid metabolism in the anti-tumor properties of TCM decoction. For instance, Liu et al. reported that Fuzheng Xiaozheng Prescription (FZXZP) exhibit satisfactory therapeutic effects in cirrhosis and hepatocellular carcinoma (HCC) treatments by activating PPAR signaling pathway [107]. Another study revealed that the therapeutic effect of Sanhuang Xiexin Decoction (SXD) on TNBC is attributed to the modulation of lipid metabolism via JAK2-STAT3 signaling suppression [108]. Untargeted lipidomic was used to analyze the serum lipid profiles of Chaihu Shugan San (CSS)-treated mice, indicating that the levels of glycerophospholipids and sphingolipids were significantly altered by CSS treatment [109]. Furthermore, a recent study demonstrated that Fuling Granule can regulate lipid metabolism to promote ovarian cancer cell migration [110]. Though ample evidence substantiates that herbal medicines can inhibit the tumorigenesis by regulating lipid metabolism, further mechanisms remain unrevealed.

In light of the multi-target features of Chinese herbs and herbal extracts, many researchers further investigated the role of TCM in the lipid reprogramming of immune cells. Ming's lab explored the potential mechanism of Danggui Liuhuang decoction (DGLHD) against fatty liver in vivo, showing that DGLHD could change PI3K/Akt signaling pathway and elevated PPAR-γ expression in DCs and T cells [111]. Another study by Mao et al. showed that Huangqin Decoction (HQD) regulate M2 macrophage polarization by activating FFAR1/FFAR4-AMPK-PPARα pathway in ulcerative colitis [112]. However, few investigations of TCM decoction on the lipid reprogramming of cancer-related immune cells was found.

### 4.2 Bioactive compounds from TCMs modulate lipid metabolism of cells within tumor microenvironment

Previous reviews indicated that natural products derived from TCM suppressed fatty acids biosynthetic pathway by targeting metabolic enzymes and were regarded as promising inhibitors for cancer treatment [113]. For instance, FABPs, a family of transport proteins for fatty acids, were frequently reported overexpressed in cancer cells. The natural compound GL22, extracted from Ganoderma mushrooms, was reported to induce mitochondrial dysfunction, decreased ATP production, and cell death of hepatocellular carcinoma via the inhibition of FABPs expression [114]. The ethanol extract of Polygoni multifori Radix exhibited the anti-proliferation property against hepatocellular carcinoma by inhibiting the production of unsaturated fatty acids, which is mediated by SREBP-1, a master transcriptional regulator of lipogenic gene [115]. Moreover, many active components derived from Chinese herbs displayed FASN inhibitory activity in various types of cancers, such as quercetin, osthole, and oridonin [[116], [117], [118]]. Epicatechin gallate, which is extracted from green tea, has been found to significantly reduce the synthesis of fatty acids and inhibit cell proliferation in prostate cancer. This effect is achieved by reducing the expression of two enzymes: acetyl-CoA carboxylase (ACC) and ATP citrate lyase (ACLY) [119]. Likewise, Emodin was reported to restrain the growth of human hepatocellular carcinoma cells via the decreased cholesterol biosynthesis and SREBP2 transcriptional activity [120]. Protopanaxadiol and carnosic acid, derived from ginseng and rosemary respectively, have both exhibited inhibitory effects on cellular cholesterol synthesis in colon cancer cells [121,122].

In TME, immune cells display increased immunosuppressive effects by regulating their lipid metabolism patterns, and subsequently promoting tumor progression [123]. Consequently, altering the lipid metabolism patterns of immune cells may be a potential therapeutic strategy to modify the immunosuppressive TME, which subsequently blocks tumor progression. Saikosaponins-a (SSa), a major bioactive extract of Radix Bupleuri, has been shown to obviously reduce lipoprotein uptake via fatty acid receptor CD36 in THP-1 cells, leading to impaired immune response [124]. A study of the effect of Dioscin against colitis suggests that Dioscin could promote FAO of macrophage via regulating the mTORC2/PPAR-γ signals [125]. Though documented literatures have clarified the molecular and functional interplay between lipid metabolism and immune cell biology, there still has a long way to go to develop it into therapeutic intervention for neoplasms.

### 4.3 TCMs and its bioactive compounds modulate lipid profiles of mouse and human *in vivo*

As TCM can regulate all steps of lipid metabolism, there is sufficient clinical evidence that TCM is involved in the regulation of lipid metabolism in the treatment of diseases highly related to lipid metabolism [126]. An analysis of real-world data shows that botanical drugs regulate glucose and lipid metabolic pathways, thereby alleviating the decline of renal function in patients with hypertensive nephropathy [127]. Meta-analysis of randomized controlled trials shows that Chinese herbal medicine as an add-on therapy for type 2 diabetes mellitus patients with carotid atherosclerosis has more advantages than western medicine alone, because Chinese herbal medicine can further regulate the glucose and lipid metabolism of patients [128]. Likewise, a clinical study on cinnamon, a traditional Chinese medicine, suggest that cinnamon may be utilized as lipid-lowering supplement in clinical settings with a guaranteed safety profile [129]. Additionally, a clinical data collected from 1990 to 2016 showed that 282 TCMs have the potential to regulate lipid metabolism. According to their mechanism of action, they can be roughly divided into three categories: inhibiting synthesis, increasing decomposition, and reducing absorption [130].

Some researchers evaluated the lipid-lowering effects of active extracts from TCM. Lin et al. [131] investigated the effect of hawthorn extracts of Crataeguspinnatifida on cholesterol metabolism in animal model, showing that hawthorn extract lowers plasma non-HDL-C by 8 % without changing HDL-C. Further investigations indicated that Oleanolic acid (OA) and ursolic acid (UA) from hawthorn extracts could reduce intestinal cholesterol absorption via inhibition of intestinal ACAT2 activity [131]. Similarly, treatment with Berberine in rats on atherogenic diet was found to lowers blood TC and non-HDL cholesterol levels partly through inhibition of intestinal ACAT activity [132]. Extensive clinical studies have verified the therapeutic role of ginseng and ginsenosides in multiple metabolic syndromes, including obesity, diabetes, dyslipidemia, or non-alcoholic fatty liver disease [[133], [134], [135], [136]]. In ginseng-treated mice, serum lipid profiles inclusive of triglycerides, total cholesterol, free fatty acids were dramatically altered [134]. In a preclinical study, serum triglycerides level (302.0 ± 70.4 vs. 527.7 ± 153.3 mg/dL) was found to be reduced in the KRG-administered group when compared with NAFLD group (p < 0.05). In addition, HDL cholesterol levels (liver tissue, 4.8 ± 0.2 vs. 4.2 ± 0.2 mg/g) were elevated when compared to the NAFLD group (p < 0.001) [136]. Taken together with above experimental and clinical data, TCM has clear evidence to regulate lipid metabolism in vivo. The collected information above is summarized in Table 3.

**Table 3:** TCMs and its bioactive compounds interfere with lipid reprogramming within TME.

TCMs and its bioactive compounds	Metabolic disorders	Targeting metabolic/metabolic enzymes/pathways within TME	Reference
Dioscin	non-alcoholic fatty liver disease, colitis	SIRT1/AMPK signal pathway, mTORC2/PPAR-γ mediated FAO in macrophage	[104,125]
Berberine	cardiovascular diseases, Rats on atherogenic diet	LDLR/ERK/JNK or LDLR/(MAPK)/ERK pathway, lowers blood TC and non-HDL cholesterol levels via ACAT activity inhibition	[105,132]
Curcumin	dyslipidemia	AKT/mTOR pathway	[106]
Fuzheng Xiaozheng Prescription	hepatocellular carcinoma	PPAR signaling pathway in cancer cells	[107]
Sanhuang Xiexin Decoction	Triple-Negative Breast Cancer	JAK2-STAT3 signaling suppression in cancer cells	[108]
Chaihu Shugan San	prostate cancer	glycerophospholipids and sphingolipids in cancer cells	[109]
Fuling Granule	ovarian cancer	fatty acid in cancer cells	[110]
Danggui Liuhuang decoction	Hepatic Steatosis	PI3K/Akt signaling pathway and elevated PPAR-γ expression in DCs and T cells	[111]
Huangqin Decoction	ulcerative colitis	FFAR1/FFAR4-AMPK-PPARα pathway in M2 macrophage	[112]
GL22	hepatocellular carcinoma	FABPs expression in cancer cells	[114]
The ethanol extract of Polygoni multifori Radix	hepatocellular carcinoma	SREBP-1 expression in cancer cells	[115]
quercetin, osthole, and oridonin	cancers	FASN expression in cancer cells	[[116], [117], [118]]
Epicatechin gallate	prostate cancer	ACC, ACLY expression in cancer cells	[119]
Emodin	hepatocellular carcinoma	cholesterol biosynthesis and SREBP2 transcriptional activity in cancer cells	[120]
Protopanaxadiol, carnosic acid	colorectal cancer	cholesterol synthesis in cancer cells	[121,122]
Saikosaponins-a	atherosclerosis	CD36 in THP-1 cells	[124]
Cinnamon	T2DM diabetes	glycolipid metabolism	[129]
Hawthorn extracts of Crataeguspinnatifida	Hamsters on high-fat diet	HDL-C	[131]
Oleanolic acid, ursolic acid	Hamsters on high-fat diet	cholesterol absorption via inhibition of intestinal ACAT2 activity	[132]
Ginseng, ginsenosides	old-aged ob/ob mice	triglycerides, total cholesterol, free fatty acids	[[133], [134], [135], [136]]

## 5 Perspectives and conclusions

Lipids, as the principal constituents of cell membranes and vital bioactive molecules in cellular processes, play a crucial role in the adaptive alterations of tumor cell metabolism and are closely associated with malignant biological behaviors such as tumor invasion and metastasis. The reprogramming of lipid metabolism in tumor cells represents a common and pivotal metabolic characteristic in the course of tumor evolution, enabling their survival and further progression in hostile environments. Importantly, immune cells in the tumor microenvironment (TME) also undergo abnormal lipid metabolism, and the tumor metabolic reprogramming affects the functional state of immune cells in the tumor immune microenvironment, further promoting invasion and metastasis. Nonetheless, the connection between aberrant tumor lipid metabolism and tumor immunity still faces numerous challenges. Firstly, the incomplete understanding of the molecular mechanisms underlying tumor lipid metabolism reprogramming poses a major obstacle in the development of anti-tumor therapies targeting lipid metabolism alterations. Secondly, the complex interplay between oncogenic signals and lipid metabolism requires a more comprehensive understanding of lipid alterations in tumors. Moreover, the distinct lipid profiles in tumor patients reflect diverse metabolic adaptation strategies of tumor cells, necessitating further research to elucidate the potential mechanisms and significance of plasma lipids in tumor molecular features and disease progression. Lastly, additional studies are necessary to acquire full comprehension of the molecular mechanisms underlying the metabolic changes and immune evasion within TME.

As we reviewed here, amounting evidence have indicated the close links between lipid metabolism and tumor onset, progression, metastasis, and immune evasion. However, we are only beginning to the role of lipid metabolism in separate cell types within TME, especially in vivo. Amounting fundamental concerns remain uncovered, including the role of lipid metabolism and lipid metabolites in the crosstalk between cancer cells and immune cells. For example, how do lipids of cancer cells interplay with signaling pathways to induce immunosuppressive TME? Do lipids from different cellular sources or compartments have distinct signaling roles? Further, whether and how the metabolic plasticity of lipid metabolism allows immune cells to adapt to environmental changes (e.g., the availability of various nutrients) and sustain their immunogenic activity in different contexts are also an interesting question to explore.

Amounting papers have confirmed that TCM can effectively intervene in tumor metabolism through multiple targets and pathways, inhibiting tumor cell proliferation and promoting tumor cell apoptosis, thereby achieving the effect of tumor suppression. Integrating TCM with current cancer treatment methods may provide novel ideas and strategies for current tumor treatment. However, existing studies mainly focus on the exploration of the regulatory effects and mechanisms of TCM monomers on tumor metabolism, with relatively fewer studies on the regulation of tumor metabolism by TCM formulations. TCM formulations have the advantages of multiple components and multiple targets, demonstrating prominent efficacy in clinical tumor treatment. Nevertheless, the complexity of their drug composition and various influencing factors may pose multiple challenges to research. Therefore, the exploration of the mechanisms of TCM formulations in intervening in tumor metabolic reprogramming will forge a path toward improved therapies and outcomes for cancer patients.

## Funding

This research was supported by the 10.13039/501100001809National Natural Science Foundation of China (81974571, 82274513 and 82305234) and Guangdong Natural Science Research Grant (2020A1515110760 and 2023A1515011115). This study was also supported by the Guangdong Hospital of Traditional Chinese Medicine Special Research Project on Traditional Chinese Medicine Science and Technology (YN2022QN32).

## Institutional review board statement

Not applicable

## Informed consent statement

Not applicable.

## Data availability statement

No data was used for the research described in the article.

No additional information is available for this paper.

## CRediT authorship contribution statement

**Qian Zuo:** Writing – original draft, Funding acquisition, Conceptualization. **Yingchao Wu:** Writing – original draft. **Yuyu Hu:** Validation, Conceptualization. **Cui Shao:** Writing – original draft. **Yuqi Liang:** Visualization. **Liushan Chen:** Investigation. **Qianqian Guo:** Formal analysis. **Ping Huang:** Project administration. **Qianjun Chen:** Writing – review & editing, Funding acquisition.

## Declaration of competing interest

The authors declare the following financial interests/personal relationships which may be considered as potential competing interests: Qianjun Chen reports article publishing charges was provided by National Natural Science Foundation of China. If there are other authors, they declare that they have no known competing financial interests or personal relationships that could have appeared to influence the work reported in this paper.
